# First Dental Steps intervention: feasibility study of a health visitor led infant oral health improvement programme

**DOI:** 10.1186/s12903-025-06154-4

**Published:** 2025-07-03

**Authors:** Joanna G. Williams, Patricia Nicole Albers, Sarab El-Yousfi, Zoe Marshman, Reena Patel, Alice Porter, Katrina d’Apice, Katie Breheny, Frank de Vocht, Chris Metcalfe, Robert Witton, Ruth Kipping

**Affiliations:** 1 Bristol Medical School, Population Health Sciences, University of Bristol, Bristol, England; 2 School of Clinical Dentistry, University of Sheffield, Sheffield, England; 3 NHS England South West, Taunton, England; 4 NIHR ARC West, Bristol, England; 5 Faculty of Health: Medicine, Dentistry and Human Sciences, University of Plymouth, Plymouth, England

**Keywords:** Oral health, Dental, Dental caries, Health visiting, Public Health nursing, Children under 5-years

## Abstract

**Background:**

In England, dental caries is common (22.4% of 5-year-olds, 2024) and the primary reason for hospital admission of children. First Dental Steps (FDS), an intervention in South West England, includes training for health visitors, integrating oral health advice into home visits and provision of oral health packs (a free flow cup, toothbrush and 1450ppm fluoride toothpaste) to vulnerable families at the 1-year developmental check. The aim was to conduct a feasibility study of FDS to support parents to increase infant toothbrushing.

**Methods:**

This study explored the feasibility and acceptability of the FDS intervention and research methods in 5 local authority areas in South West England and 4 comparison sites. Data collection (June 2021-February 2022) included baseline and follow-up questionnaires (mean 5 months), semi-structured interviews with parents (*n* = 16), health visitors (*n* = 7), and stakeholders (*n* = 16). Analysis of questionnaires was descriptive, and interviews were analysed using the framework method.

**Results:**

Parents completed baseline (*n* = 59) and follow-up questionnaires (*n* = 26), with 10 parents (40%) reporting an increase in brushing frequency and 4 families (15%) reported visiting a dentist. From the interview data, five themes were identified 1) acceptability of the intervention, 2) feasibility of the intervention (delivery and implementation), 3) possible benefits of the intervention, 4) acceptability of study methods, and 5) suggested improvements. The FDS intervention was seen to be acceptable and feasible to those who took part, however, challenges with recruitment and retention demonstrate that obtaining data for a full trial would not be feasible.

**Conclusions:**

Parents, health visitors and stakeholders who took part in our study found FDS to be acceptable and feasible. Recruitment, retention and study methods (completing participant flow table) were challenging, and the progression criteria for the research methods were not met to progress to a full trial. Modifications were recommended to improve the intervention and further co-production approaches could be used to ensure it is culturally appropriate in a diverse population.

**Supplementary Information:**

The online version contains supplementary material available at 10.1186/s12903-025-06154-4.

## Background

Globally, untreated dental disease is the most widespread health challenge, with poor and vulnerable groups being most affected [1]. Dental caries in childhood has been linked to poor dental health in adulthood [2]. In a recent English national survey, 22.4% of 5-year-old children had experienced obvious dental decay [3]. In England, dental caries in young children is the primary reason for hospital admissions [4], which is upsetting and disruptive for children and families [4] as well as placing a financial burden on the National Health Service (NHS). Improved oral hygiene and early management of dental caries, including regular toothbrushing with fluoride toothpaste, reduction of sugary food and drinks, and regular dental check-ups, can prevent tooth decay and extraction [5, 6].

It is well established that health status and behaviours differ by socio-economic status, and this is also true for oral health and oral health behaviours [7]. The link between deprivation and poor oral health has been evidenced and understanding these mechanisms is important to improve targeted service delivery and policy [7]. It is believed that socio-economic status and ethnicity influence oral health by driving oral health behaviours, specifically oral hygiene, diet, smoking, and visiting a dentist [8], these inequalities are especially pronounced in young children. Social gradients exist in child oral health and often remain into adolescence; a key driving factor is residential deprivation [8]. Moreover, social inequalities in the prevalence of dental caries in 5-year-old children have increased from 2008 to 2019 [9].

National guidance for England recommends specific oral health initiatives to improve oral health and reduce oral health inequalities [10], one of these initiatives is provided via the Healthy Child Programme albeit, oral health training is not obligatory [10]. The under 5 years of age component of the Healthy Child Programme is commissioned primarily through a health visitor provider, working alongside other early years providers [11]. In the UK, health visitors are specialist community public health nurses or midwives who support the health and development of infants and children until they are 5 years old, these teams work closely with families by providing advice and support. There are five mandated family visits that health visitors undertake, namely; antenatal visit (28 weeks of pregnancy), new baby review (10–14 days after birth), 6-8 week review, 9–12 month development review, 2–2 $\frac{1}{2}$ year development review [11]. There are also additional non-mandatory reviews at 3–4 months and 6 months [11].

A scoping review on the role of health visiting teams in improving children’s oral health indicated health visiting teams were well placed in principle to promote oral health to young children and their families [12]. Their role involved delivering oral health advice, oral health packs and other oral health resources, as well as promoting dental registration and referral to dental services [12]. The findings of the review suggested the majority of health visitors found this role acceptable and identified the need for improved formal education, training and training resources for health visitors in oral health [12]. Additionally, the findings identified several barriers faced by health visitors in promoting children’s oral health such as; limited resources with consequent competing priorities, lack of confidence in providing oral health advice due to inadequate training and communication barriers with both parents and dental services [12]. Similarly, a recent study [13] exploring the acceptability of a health visitor-delivered intervention for children aged 9–12 months found that both parents and health visitors perceived the provision of oral health advice as important. Additionally, health visitors noted the need and importance of training and the challenges around delivering oral health messages in the face of competing priorities [13].

However, there are limitations in the existing evidence to support such community-based oral health interventions and further research is needed [14, 15]. This feasibility study sought to address this gap by exploring the feasibility and acceptability of the First Dental Steps (FDS) Intervention [16]; a health visitor delivered oral health intervention to parents of 12-month-old children. The FDS intervention was embedded into the Healthy Child Programme, enabling oral health improvement to be integrated with other health objectives, such as nutrition and healthy weight guidance.

In this study we aimed to assess the delivery of the FDS intervention and uncertainties related to the acceptability, recruitment, and retention of participants, to inform the design of a possible cluster randomised controlled trial (RCT). Six objectives were identified to support this aim:

1. To explore the feasibility of delivering the FDS intervention including different delivery methods and barriers and facilitators to implementation
2. To explore the acceptability of the FDS intervention to health visitors and parents
3. To determine the likely recruitment of parent study participants at baseline and retention at follow-up
4. To determine if the study methods were acceptable to health visitors and parents
5. To explore the possible effect of the intervention on toothbrushing
6. To pilot and refine methods and resource use data collection to estimate intervention costs and consequences in a future RCT

## Methods

**Fig. 1:**
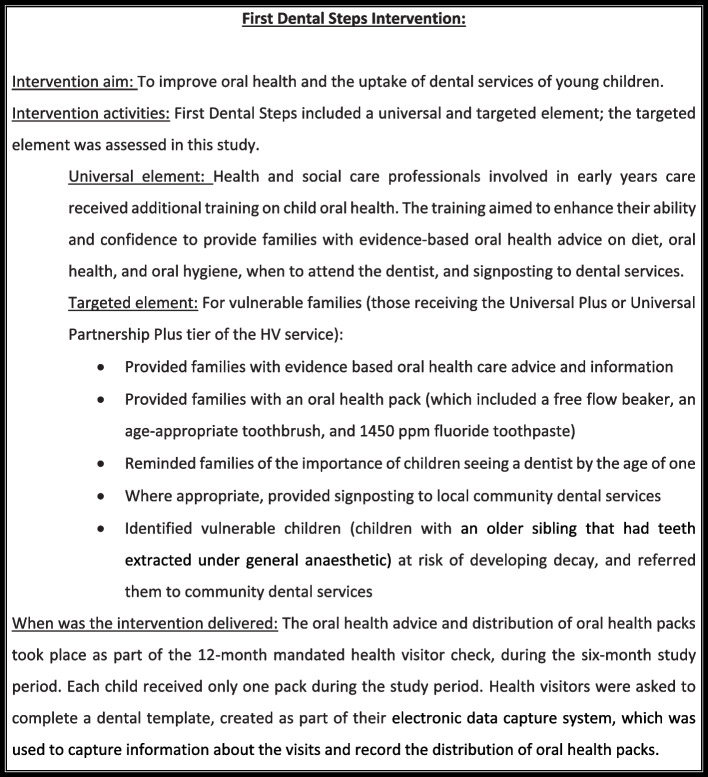
First Dental Steps Intervention

The study was a mixed-methods feasibility study of the FDS intervention. Figure 1 provides a summary of the FDS Intervention. The introduction of this initiative in local authorities (LAs) in South West England provided the opportunity to explore the feasibility and acceptability of this intervention.

This feasibility study was originally conceptualised prior to the COVID-19 pandemic. There were significant disruptions to the health visiting services at the onset of the pandemic and as a result, we redesigned aspects of the feasibility study in collaboration with health visiting teams. Some of these adaptations included accounting for non-face-to-face reviews, posting of oral health packs, and using digital data collection options that could be accessed via mobile phone. Further details of the intervention and study methods can be found in the protocol paper [16].

### Sample and Recruitment

Five of six LAs, purposively selected by Public Health England to deliver the FDS intervention, were invited to participate. Intervention sites began delivering the intervention prior to recruitment, ensuring their familiarity with the process before adding the research element.

Four additional non-randomly selected LAs (from South West England) not involved with FDS or any other child oral health interventions, were invited to participate as comparison sites. These sites only took part in stakeholder (LA oral health leads and health visiting team leads) interviews.

When delivering the FDS intervention to vulnerable families (within the intervention LA areas) the health visiting team invited these families to participate. Vulnerable families were defined as those who were identified as needing targeted (Universal Plus (UP)) or specialist (Universal Partnership Plus (UPP)) support. The targeted level of service (UP) would provide support for instance with postnatal depression, weaning, or child sleep challenges. The specialist, multiagency UPP level of service is provided for vulnerable children and families needing ongoing support, often those with complex or additional health needs. Families provided consent to complete a baseline and follow-up questionnaire and gave additional consent for an optional telephone interview. No other inclusion or exclusion criteria were applied.

Across the five intervention sites, an estimated 789 vulnerable families were expected to be seen during the six-month study period (01 June 2021 to 31 January 2022).

#### Recruitment for interviews

LA oral health leads and health visiting team leads across all the intervention and comparison LA areas were invited for an interview. Health visitors, involved in the delivery of the intervention, were invited. We planned to use purposive sampling for all interviews, however, with fewer responses than anticipated we instead interviewed all those who agreed.

### Patient and Public Involvement and Engagement (PPIE)

A dedicated public involvement and engagement group, which included primarily parents of young children, was established. This group was consulted about the usability and understandability of the baseline and follow-up questionnaires, parent information letter, consent form, and topic guides.

### Outcome measures

#### Effectiveness outcomes and secondary outcomes

Feasibility and acceptability were assessed using interviews, applying semi-structured topic guides with additional scope for probing by the interviewer. Topics included interviewees’ experience of the intervention, how well it aligned with the content of the visit, whether it was appropriately timed, whether there was sufficient time during the mandated family visit, thoughts on the oral health packs, improvements, and whether the intervention should continue. We also explored recruitment and retention rates, facilitated by a SPIRIT checklist [17], hereafter referred to as the *participant flow table* (see protocol paper for further details [16]). Health visiting teams recorded and tracked participant movement through the study, and were also asked to record invited families that declined involvement.

Secondary outcomes included oral health behaviours such as toothbrushing frequency, toothpaste use, diet (specifically sugar consumption) and self-reported dental check-ups.

#### Opportunities for resource use data collection

Where possible intervention costs, including the cost of the oral health packs and resources associated with training, were collected. During the health visitors interviews, we explored time needed for intervention delivery. Families were asked if they incurred any additional costs by independently continuing FDS intervention behaviours.

### Data collection

#### Parental survey

Baseline and follow-up data were collected using a self-completed digital questionnaire. A short, 9 item, questionnaire was designed based on existing questionnaires [18–22] and tailored with the support of the PPIE group. These were completed online via REDCap, a secure data capture system hosted by the University of Bristol, with the assistance of a health visitor, if needed. The questionnaire covered current toothbrushing practices, diet, dental check-ups, and demographic details.

#### Parental and stakeholder interviews

Participating parents were invited to an interview after they had received the intervention (between July 2021 and November 2021). All parents received a £10 shopping voucher for their participation.

Interviews were conducted with all agreeing stakeholders (LA oral health leads and health visiting team leads). Additionally, for the intervention sites, health visitors involved in the intervention and study were invited to an interview. Interviews were conducted between July 2021 and February 2022, over the telephone or video conferencing software and lasted between 30 and 60 min. All interviews were audio recorded and professionally transcribed verbatim and anonymised for analysis. See Additional File 1 for all interview topic guides.

#### Resource use

Training workshop costs were estimated by recording the number of attendees, their roles, location and the mode of delivery (face to face or online). Personal Social Services Research Unit (PSSRU), Unit Costs for Health and Social Care (2021) were used to estimate the cost of staff time to attend [23]. Travel costs, estimated pragmatically using one distance between training location and health visitor base, were costed using the HMRC approved mileage and fuel rate (£0.56 per mile) [24]. Oral health pack procurement was provided by Public Health England (£1.92 unit cost and £12.00 delivery per batch). It was assumed that all packs were distributed to families with no wastage. Family and health visitor interviews included questions exploring potential public health sector and societal costs. Later in the study, PPIE representatives reviewed a potential resource use questionnaire for feasibility of use in a future RCT.

### Data analysis

#### Parental survey data

Descriptive analysis (mean and standard deviation, or number and percentage) of consent, recruitment, response rates, and loss to follow-up of parents were explored, and are presented by site (Table 1). Descriptive summaries of demographic details and oral health behaviours are presented. The change in frequency of reported oral health behaviours between baseline and follow-up is presented.

**Table 1:** Recruitment and retention rates by site

	Consent to Study*	**Baseline**	**Follow-up (T1)#**	**Consent to Interview**	**Completed Interview**
*All*	*69*	*59 (85.5%)*	*26 (44.1%)*	*57 (82.6%)*	*16*
Site 1	8	5 (62.5%	4 (80%)	5 (62.5%)	1
Site 2	6	6 (100%)	1 (16.7%)	5 (83.3%)	1
Site 3	6	5 (83.3%)	1 (16.7%)	6 (100%)	0
Site 4	4	4 (100%)	NA	1 (25%)	0
Site 5	45	39 (86.7%)	20 (51.3%)	39 (86.7%)	14

^*^ Key Progression criterion: > 30% parental consent rate for eligible children

^#^ Key Progression criterion: < 30% parental loss to follow-up

#### Parent and stakeholder interviews

All interview data were analysed using framework analysis [25], a deductive-inductive hybrid approach was used permitting the exploration of the research questions and themes identified a priori, while allowing new themes to be identified.

KD and SE randomly selected and independently coded two parent interview transcripts. Codes were discussed, grouped into categories, and the coding framework was agreed upon. KD coded and analysed the remaining parent transcripts: the coding framework was applied, a framework matrix created, and each cell summarised. For each summary, elements were detected and grouped into key underlying dimensions. Key dimensions were categorised into sub themes, and sub themes grouped into themes. All stakeholder interview transcripts were double coded by AP and SE, following the above procedure. All coding was conducted in NVivo (version 1.5.2). Emergent themes from the parent and stakeholder interviews were similar and as a result, we merged themes and data for parents and stakeholders are reported under these.

#### Resource use

Costs of training workshops and oral health packs are presented using descriptive statistics. Qualitative data regarding resource use were summarised by KD, AP and SE.

## Results

### Recruitment and retention at follow-up

#### Parents

Recruitment and retention rates are provided in Table 1. In total 69 participants consented. Recruitment was low across all but one site (Site 5). Furthermore, this site was the only site able to reach their estimated recruitment number. Retention at follow-up varied by site, but overall was relatively low with less than half of the parents completing the follow-up questionnaire. 57 parents (83%) consented to the telephone interview, but only 16 completed the interview. The majority of parents (n = 14) resided in Site 5, and the remaining two parents lived in Sites 1 and 2.

The participant flow tables were not well completed by all sites, with few entries for families who declined participation and limited capture of the follow-up process. Based on available data, health visitors being unable to reach the families was the primary reason for loss to follow-up. Given that sites struggled to complete these tables the inclusion of them was not feasible.

#### Stakeholders

In total, 23 stakeholder interviews were completed (Table 2) 21 were conducted with health visitors, health visitor team leads, and LA oral health leads. We also interviewed two of the training providers. We were unable to recruit any staff from Sites 7 and 8.

**Table 2:** Breakdown of stakeholder interviews

	**Health Visitors**	**Health Visitor Team Leads**	**LA Oral Health Leads**
**All**	**7**	**7**	**7**
Intervention sites			
Site 1	2	1	1
Site 2	1	1	1
Site 3	2	1	1
Site 4	1	0	1
Site 5	1	2	1
Comparison Sites			
Site 6	NA	1	1
Site 7	NA	0	0
Site 8	NA	0	0
Site 9	NA	1	1

2 × Training providers

### Questionnaire data

#### Demographics

The mean age of the children at baseline was 10 months (range 9 to 14 months) and 15 months at follow-up (range 12 to 17 months). Most respondents (97%) were the mother or father of the child and the majority reported their ethnicity as white (98%). Over half (56%) reported that the child had an older sibling.

Of the families (69), 46% (*n* = 32) were classified as Universal Plus and 32% (n = 22) were classified as Universal Partnership Plus. UP or UPP classification were not available for 22% (*n* = 15) of families. Four families were recorded as having a sibling that had teeth extracted under a general anaesthetic.

#### Toothbrushing

At baseline, 53% (*n* = 31) of parents reported brushing their child’s teeth twice a day. Whereas 25% (*n* = 15) reported brushing their child’s teeth less than once a day. Of the 26 parents that completed both the baseline and follow-up questionnaires, 39% (*n* = 10) reported brushing their child’s teeth twice a day and 30% (*n* = 7) reported brushing their child’s teeth less than once a day. At follow-up, this increased to 73% (*n* = 19) of parents reporting brushing their child’s teeth twice a day.

#### Visiting a dentist

At baseline, of all the families, 9% (n = 5) reported visiting a dentist either to get their child used to going or for a check-up or treatment. Of those that completed both time points 8% (n = 2) visited a dentist at baseline compared to 23% (n = 6) at follow-up.

#### Cup type

It was evident that fewer children were using baby bottles at follow-up with more children using free-flow beakers (Table 3). Children’s diets also changed over the five months, with more children consuming sugary drinks and food at follow-up.

**Table 3:** Cup type at baseline and follow-up

	**Baseline: full sample (*n* = 59)**	**Both time points (*n* = 26)**	
		**Baseline (T0)**	**Follow-up (T1)**
Baby bottle	48% (n = 28)	50% (n = 13)	15% (*n* = 4)
‘Sucky’ beaker	28% (n = 16)	19% (n = 5)	42% (*n* = 11)
Free flow beaker or open cup	4% (n = 12)	23% (n = 6)	42% (*n* = 11)

### Stakeholder and parent interviews

Qualitative analysis of the interviews with parents and stakeholders identified five themes; 1) acceptability of the intervention, 2) feasibility of the intervention (delivery and implementation), 3) possible benefits of the intervention, 4) acceptability of study methods, and 5) suggested improvements.

#### Acceptability of the FDS intervention

Parents found the oral health advice easy to understand. Some parents thought the advice was quite basic whereas others found it comprehensive. Parents reported that the advice varied according to their needs such as not being first time parents.

The oral health packs were generally appreciated by parents, even if they had already purchased the items separately. Additionally, parents mentioned the oral health pack items were more suitable for their child than items they had already purchased. Parents valued having professional guidance on suitable oral health items.

“*It’s nice to […] know that you are using what is advised really, and because it’s been given to me by a professional rather than having to physically seek it out myself and potentially do damage instead of good*.” – Parent 11

However, the perceived usefulness of the intervention depended on parents’ prior knowledge and resources, many parents felt they were well informed and had previously purchased the items included in the oral health pack. Some parents reported the intervention had improved their confidence in caring for their child’s oral health. Generally, parents stated minimal extra costs in adhering to the intervention, where additional costs were stated, parents thought these were worthwhile to mitigate future problems.

The intervention was acceptable to health visitors, health visitor team leads, and oral health leads. They felt the intervention was simple to deliver and fitted well within the content of the 9 - 12 month review. The intervention aligned with health visitor priorities, as many regarded oral health to be important. Oral health leads felt the intervention aligned well with the current Healthy Child Programme to fill the gap in provision and achieve desired oral health outcomes.

*“They were happy to be involved. From the outset I think they were keen. And I think they saw it as their place to be part of this. So I don’t think there was any resistance from either the management in the health visiting service or the staff.”* – Oral Health Lead

The opportunity to upskill the workforce and provide oral health packs to parents was highly valued. Health visitors thought the oral health advice and packs were well received by parents.

*“It's been great to have this opportunity to upskill the workforce, to take part in research, to be able to provide the families with a resource as well, both information and also a physical resource in the toothbrush and the pack that we're actually offering to them.”* – Health Visitor Team Lead

Some health visitors felt that oral health was not a priority for parents until dental problems arose, and they were concerned the advice to register with a local dentist could not be realised because of the long waiting lists and widespread difficulty finding an NHS dentist.

#### Feasibility of the FDS intervention (delivery and implementation)

The intervention was delivered in person to all parents. Barriers to implementing the intervention included the child sticking out their tongue, chewing or trying to hold the toothbrush and thus some parents hadn’t developed an effective technique.

“*It’s quite difficult to hold a baby and brush his teeth, especially if he thinks it’s food*.” – Parent 04

Some parents stated that they sometimes lacked the time or motivation to brush their child’s teeth as it was a difficult or frustrating process, whereas others reported no barriers to implementation.

Most parents had not yet taken their child to a dentist, citing long waiting lists as the most common reason. This was also a common barrier stated by health visitors and health visitor team leads. They expressed frustration about the lack of NHS dental services seeing new patients because of the backlog caused by the COVID-19 pandemic. This meant that many parents could not follow the health visitors advice to take their child for a dental check-up by the age of one year. Health visitors felt this was out of their control.

*“I started going to register her and the dentist that I’m registered with just turned around and said, “Not until she’s two.” Because I said to her [the health visitor], “I’m disgusted, you told me to go and sort it out.” Which, being a mother, or a father, in their rights would go and do that. And just being chucked away, it’s just not right, regardless of whether she has teeth yet or not.” –* Parent 12

*“It makes me feel like I'm banging my head against a brick wall sometimes, because you can give all the advice you like, and all the health promotion, and encourage the teeth brushing twice a day, and supervised brushing until seven, but it's really difficult for people to access an NHS dentist” –* Health Visitor

However, availability at dentists did vary, some parents had successfully booked appointments for their child. One parent had chosen to wait despite the health visitors’ advice, whilst others seemed unsure of what age to take their child to the dentist.

Lack of time and the need to prioritise other issues was cited as a barrier to implementing the intervention by health visitors, acknowledged by health visitor team leads and oral health leads.. Health visitors often felt they had a lot of information to cover during visits (which usually only last 45–60 min) but were also conscious of not wanting to overwhelm parents with too much information.

*“We’ve got so much to get done. That’s why I say a minute, two minutes for dental and then you have to move on in many ways to focus on something else.”* – Health Visitor

Health visitors also had to consider the home environment, for instance parents often could not dedicate their full attention to the visit because of also having to deal with their child’s/ children’s needs. In some circumstances, health visitors needed to prioritise another issue because of a parent’s situation (e.g., mental health, safeguarding), which meant oral health was either not covered in detail or not covered at all.

*“I suppose the health visitor has to take a balanced view on whether there is the time, or indeed whether it’s appropriate to talk about issues of oral health and oral health improvement when there might be, I suppose, more serious or immediate pressures on the child and within the family.”* – Oral Health Lead

The eligibility criteria for who was to receive the intervention (i.e., Universal Plus/ Universal Partnership Plus families) was mentioned as a barrier for some. Health visitors and health visitor team leads were not always clear who should receive the intervention (and particularly the oral health packs), especially when families were moving between categorisations (universal, UP, or UPP) or were deemed vulnerable in other ways such as having a low income but not classified as UP or UPP. Some felt the eligibility criteria should instead be based on social deprivation or be at the discretion of health visitors.

Facilitators to implementing the intervention for parents included establishing a routine, toothbrushing as a family so the child mirrors others’ behaviours, and choosing an appropriate place (e.g., cot or bath). Distraction was also mentioned as a good way to aid toothbrushing.

“*if there’s a bit of distraction, so either, you know, the telly or give her another toy to play with. Yes, she seems to be a lot happier to let you do it then*.” – Parent 01

In addition, health visitors were viewed as approachable, trusted advisors that reassured parents.

*“I’ve got a good relationship with my health visitor so she’s quite friendly.[…] Just that I know I’m going to be able to care of her teeth properly. Just that I’m definitely doing the right thing, using the right materials.”* – Parent 07

Health visitors, health visitor team leads, and oral health leads perceived the oral health packs to be the greatest facilitator. The oral health packs helped to remind health visitors about the oral health information to cover, facilitate the oral health conversations, and engage parents, because parents appreciate a “freebie”. The provision of the oral health pack meant that parents could also put the advice into practice straight away.

*“it promotes that conversation, so it doesn’t just feel like a telling off by the health visitor. And again, can encourage them to actually do it, that’s the other thing. And there is something about having an intervention where you want them to do something, and you provide that person with a tool to do it.” –* Health Visitor Team Lead

#### Benefits of the intervention

Although many parents were already brushing their child’s teeth prior to the intervention, some reported that they had started brushing their child’s teeth since receiving the intervention or that they had increased the frequency of toothbrushing:

“*She was just telling me to brush her teeth twice a day and after she's had some milk. So, I've been doing that*.” – Parent 02

A benefit to the health visitors was receiving specific oral health training. Health visitors, health visitor team leads, and training providers all felt the training had increased health visitor confidence to have meaningful conversations and provide solid advice about oral health. They felt there were many benefits to offering this training to health visitors, and especially felt it was valuable to offer it to all staff rather than only staff involved with the intervention target group.

*“The health visitor training is so generic it's not very… It's much more focused on theory and research in public health rather than specific topics, so it was really interesting to have the information, statistics, gory pictures that the Dental Steps training provided.” –* Health Visitor

#### Acceptability of study methods

The study methods were acceptable to parents. They reported that they had been given all the necessary information to decide if they wanted to participate in the study, and the consent form was regarded as straightforward and easy to understand.. Parents noted several reasons why they chose to take part, such as, the ability to voice their issues with registering with a dentist, and the possible widespread benefit of research.

Conversely, some health visitors stated the consent form was “cumbersome” and complex and therefore they needed to relay the information to parents in a simpler way or go through the forms with them, which took additional time.

The study questionnaire was deemed generally acceptable to parents because it was considered to be quick to complete and easy to understand.

“*It wasn’t too long. It only took me I think a couple of seconds to answer.*” – Parent 11.

However, the questionnaire was less acceptable for parents with low levels of literacy, learning disabilities, or those who had many concerns to discuss with the health visitor.

The study methods were also acceptable to health visitors, health visitor team leads, and oral health leads, who wanted to be involved and valued the research. However, they noted that recruitment of parents was difficult, because of lack of time to go through study procedures, unwillingness from parents, technical issues with accessing electronic study documents, and the perceived burden on parents that had additional challenges.

The COVID-19 pandemic delayed the start of the study, which meant some health visitors had forgotten the study details and who the intervention was for. The pandemic also put additional pressures on the service and contributed to staffing issues. However, in a few sites, teams had circulated the study flowchart (developed by the research team), which health visitors took to family visits as a reminder of the procedures and to facilitate recruitment. Having an administrator dedicated to processing study documents and coordinating the training dates aided the smooth running of the study.

*“I've been sending out regular emails to our teams to keep the momentum going around recruitment. The general feedback I've had from the practitioners is that families just don't want to do it. They haven't got time, they've got other priorities to think through. They're really grateful for the pack and the information that they've received, but they don't want another phone call…”* – Health Visitor Team Lead

Parents also speculated that others may not wish to be involved because they may be too busy, lack time, have other priorities, be tired or sleep deprived, or do not think oral health is important.

#### Suggested Improvements

Parents stated the timing of the intervention was adequate because they had prior oral health knowledge, had sought out information independently, or their child’s teeth hadn’t yet erupted. However, several noted that first time parents would benefit from an earlier intervention. Some mentioned they would have preferred the intervention earlier, prior to the eruption of their child’s teeth, weaning, or before they bought their own oral health items. They suggested the benefits of an earlier intervention included parental knowledge on the correct toothpaste to use and starting toothbrushing earlier.

“*Because up until then, I hadn’t brushed her teeth. I thought, “If I’d have known this information earlier, I would have intervened earlier*.” – Parent 07

Timing of the intervention was also frequently discussed by health visitors, health visitor team leads, and oral health leads with a perception that the timing may be too late as children’s teeth had already started to erupt by the 9 – 12 month visit, brushing should have started, and the transition away from bottles should have already occurred. Four to six months was suggested as the ideal time for the intervention. However, there is no mandated review at this age, so the 9 – 12 month review was deemed the most appropriate.

*“I would rather give out the toothbrushes before they start getting teeth, so that families have it ready for when those teeth start erupting, rather than we see them at nine months, “Have you started brushing their teeth?” “Oh no, but he’s got three.” Actually, those teeth could have been protected a long time before that, because we’re starting solids around the six-month mark.”* – Health Visitor

Parents suggested including a video demonstration of brushing technique (e.g., holding the baby whilst brushing). Several parents were dissuaded from using the provided free flow cup because it was messy, and instead advocated for a non-spill option.

Health visitors and health visitor team leads suggested including written (or electronic) material in the oral health packs, such as “dental top tips”. They felt parents needed a visual aid to reinforce the verbal advice, signpost to additional information or contacts.

*“I think it would have been good to have had a leaflet that also went with it, that supported the messages that you were delivering.” –* Health Visitor Team Lead

#### Estimated intervention costs and consequences

The intervention costs included time for the health visitor to deliver the oral health advice, delivery and procurement of the oral health packs, and attendance of the 90-min training workshops. Twenty-six workshops took place, 16 of which were face-to-face. The total estimated cost of training (including staff time and travel) across all FDS intervention sites was £45,039.21. Provision and delivery of the oral health packs was £9,544.40. Time to deliver the oral health advice was minimal (five minutes) and was integrated into a planned visit, therefore only had a small cost impact. Feasibility issues for consideration include the collection of wastage data, systematic collection of staff banding and travel time, age-appropriate resource use measures, and challenges of questionnaire burden.

Health visitors, health visitor team leads, and oral health leads discussed continuing the intervention in their services, with some mentioning plans to continue to provide the oral health advice and oral health packs to parents and use the oral health templates. However, continuation depended on a number of important factors—regular oral health training and updates; funding and budget; and views about whether the intervention should be targeted or universal. Regular training updates were thought to be important to ensure new staff could acquire the necessary knowledge and to provide a knowledge refresh for all.

*“for any new starters, they need to have something along the lines of what I delivered, just so you start them off and they’ve got that understanding. And then they could go on to having like a refresher course annually.”* – Training Provider

Health visitor team leads and oral health leads thought the intervention was cost-effective but did not have the budget to continue important elements of the intervention (oral health packs and continued training of workforce). This also fed into views on whether the intervention should be targeted or universal; stating universal provision would be best to help normalise toothbrushing in all families. Others believed a targeted approach was best to reach families with the greatest need, reducing health inequalities. However, most agreed a targeted approach would be the only realistic option within current budgets.

*“In an ideal world it would be a universal intervention, but I think I appreciate that if there were limited resources then you’d have to target the intervention where you can have greatest impact.”* – Oral Health Lead

## Discussion

### FDS intervention feasibility, acceptability, and recommended modifications

The FDS intervention was found to be feasible to deliver and acceptable to both parents and stakeholders, but the challenges of recruiting and obtaining data for a full trial was not feasible. Health visiting teams reported that they valued the FDS intervention and the delivery of the intervention did not encounter the same challenges as the research element.

The literature includes few studies of training for health visitors [12], however, one paper explicitly mentioned the need for training the early years workforce to support them with providing advice [26]. In this feasibility study, we found that health visitors welcomed the training and opportunity to refresh their knowledge, they found it enjoyable and interesting, and reported a greater degree of confidence in delivering oral health advice to families. Health visitors felt it was easy to fit in the oral health advice in their development checks, and it fitted well with other topics (e.g. weaning). These views supporte the role of health visitors in delivering oral health interventions [12].

Parents and health visitors recommended that the oral health advice is provided earlier in the children’s development (especially for first time mothers) and prior to the eruption of teeth, weaning, and purchasing items. With changes to the Healthy Child Programme and the introduction of additional health visitor contacts with families at three and six months [27], this change could be incorporated going forward. The second recommended modification was to include some accompanying resources to reinforce and expand on the messages in FDS and to provide video prompts. In particular, parents would like a video demonstration of brushing technique, and health visitors would like a written resources for the families to refer to, in keeping with findings from other studies [26].

The results presented here are in line with a similar UK study [14] where both parents and health visitors noted the importance of the provision of oral health advice by the health visitor and the challenges around oral health discussions when faced with competing priorities within the family setting [13].

### Recruitment, retention, and acceptability of study methods

Recruitment and retention to the study was challenging and progression criteria related to this were not met (see our protocol paper [16]). Given that the participant flow tables were poorly completed it is not possibly to accurately assess if our progression criteria of recruiting at least 30% of eligible parents was met. However, based on the merged estimates, across all sites, of the number of vulnerable families (> 700) that would be seen in a six-month period we can argue that four of five sites were unable to reach this recruitment target.

As the participant flow tables were poorly completed; we do not know how many families were approached but declined to participate, nor the reasons for non-participation. Furthermore, it was not possible to collect data on the reach of the intervention, and in particular, the number of families that received the intervention but did not take part in the study.

Additionally, no site was able to achieve our second key progression criterion (< 30% loss to follow-up), demonstrating that a full trial based on this design is not feasible.

Possible reasons for these challenges include the difficulties for the health visitors taking on the recruitment to the study, the pandemic context, health visitors needing to address higher priority issues with the families, large workloads and the inability to prioritise research activities. For follow-up, health visiting teams found it difficult to make contact with parents.

However, we were able to collect meaningful qualitative data on the intervention from both parents and stakeholders, including suggestions for modifications.

### Impacts on toothbrushing and other oral health behaviours

This study was not powered to investigate an impact on oral health behaviours, and there was no control group. However, we found it was feasible to collect data on self-reported oral health behaviours. Questionnaire data showed a modest improvement in toothbrushing and cup type use, in keeping with other studies [28], however these changes may be due to the children’s increased age. Health visitors reported the difficulty of sign-posting to a dentist when families were struggling to find dental practice that would take on new patients; as has been observed previously [13, 26].

### Resource use

Important cost drivers of the intervention were training and oral health packs. An important consideration in a future study would be the collection of data on wastage and family health care resource use. This could lead to a fuller understanding of return on investment. This is important as toothbrush distribution schemes are expected to have a significant return on investment; for each £1 spent on targeted provision of oral health packs by health visitors the return on investment is £4.89 after five years and £7.34 after ten years [29].

### Strengths and limitations

The study design was co-produced with health visiting teams and with a PPIE parent group. Having been planned prior to the COVID-19 pandemic, taking this co-production model, we were able to adapt it to enable flexible approaches to data collection in the pandemic context. Additionally, FDS was a targeted intervention given only to families that were classified as vulnerable as they were receiving UP or UPP health visiting services.

It was not possible to randomise LAs to the intervention, as the decision to deliver FDS predated the start of the study. Within the context of the COVID-19 pandemic a pragmatic decision was taken not to attempt full data collection in comparison LAs due to the additional burden on the health visiting teams. Instead, limited comparison data in the form of stakeholder interviews were collected.

The ethnic diversity of parents did not reflect that of the local communities and there were no male interviewees.

## Conclusion

Recruitment to the study was low and there was a significant loss to follow-up, therefore the progression criteria [16] for a future RCT were not met. The FDS intervention was acceptable to those who took part in our study including parents, health visitors, and other stakeholders including oral health leads and commissioners and leads of health visitor services.

Study methods presented challenges, and would need to be adjusted in future studies by shifting the research tasks to embedded research staff within the health visiting teams. For example it was too burdensome for health visiting teams to be responsible for various aspects of the research process which included recruiting participants, supporting families with completing consent forms and the questionnaire, completing the participant flow table, and contacting families at follow-up. Families reported that the questionnaire was very quick and easy to complete, however, some reported that the consent process was cumbersome. Future studies should explore methods to simplify and make the consent process more accessible.

Delivery of FDS was shown to be feasible, but future delivery within the Healthy Child Programme should take account of suggested modifications which include providing advice and the oral health pack at an earlier contact with the family, such as at three or six months. This is to ensure families receive the advice prior to the eruption of teeth, weaning, and purchasing of items. Additionally, the incorporation of visual and written aids such as video demonstrations on how to brush a baby’s teeth or very simple, graphic leaflets with the key messages that could be provide or shown to families during the visit. To meet the needs of ethnically diverse populations [30], appropriate modification to delivery should be co-produced with families and stakeholders.

## Supplementary Information

Additional file 1. Qualitative interview topic guides (parents, health visitors, health visiting team leads, and LA oral health leads.

## Data Availability

No datasets were generated or analysed during the current study.

## Abbreviations

FDS: First Dental Steps
LA: Local Authority
NHS: National Health Service
PPIE: Patient and Public Involvement and Engagement
RCT: Randomised Controlled Trial
