# Supplementary material for: First Dental Steps intervention: feasibility study of a health visitor led infant oral health improvement programme
Source: BMC Oral Health. 2025 Jul 3;25:1087. doi: 10.1186/s12903-025-06154-4 (PMC12231303; doi:10.1186/s12903-025-06154-4)
Supplement: Supplementary file 1 — Additional file 1. Qualitative interview topic guides (parents, health visitors, health visiting team leads, and LA oral health leads. [file 12903_2025_6154_MOESM1_ESM.docx]

**Additional File 1.**

Qualitative interview topic guides for a) parents, b) health visitors, c) stakeholder (health visiting team leads, Local Authority oral health leads, and training providers).

1. Parents

**First Dental Steps Intervention - Feasibility Study**

Interview Topic Guide - Parents

*Flexibility should be used when undertaking the interviews and applying the topic guide in terms of wording of questions, order of questions, use of probes/prompts and every opportunity made to allow participants to raise their own issues.*

**Opening:**

- Thank you for participating.
- Few things to run through before we start:
  - Confidentiality – information collected during the study is confidential and access will be restricted to our research team. Some of your comments may be included in a report on the study or in articles for scientific journals but these will be completely anonymous.
  - There are no right or wrong answers, it is your opinion that is valued.
  - Should a safeguarding issue be raised, we will follow standard safeguarding procedures and refer the concern to the relevant Health Visitor team or relevant authority such as Social Services.

**TURN ON AUDIO RECORDER**

- Consent.
- What did you think of the information you received in the health visit regarding taking care of your child’s teeth?
- Did you feel that the information you were given was clear and easy to understand?
- From the information that was given to you by the health visitor, was there anything that you found particularly useful?
- Can you tell me a little bit about the advice that was given to you by the Health visitor?
  - Probe: The topics in general?Any specific advice that you remember?
  - Toothpaste concentration?
  - When to start brushing?
  - Drinking from a cup rather than a bottle?
- Did you feel you were able to ask questions about your child’s oral health?
- Did you feel that all your questions about your child’s oral health were answered?
- What are your thoughts on the timing of the intervention?
  - Probe: Do you think the information and support given was provided at the right time?
  - Would you have preferred it earlier?
- What did you think of the of the oral health pack?
  - Probe: The contents? The logo?
  - What did you like most?
- Do you feel that the intervention has helped you take care of your child’s teeth?
- What has been your experience of toothbrushing with your child?
  - Probe: Have you experienced any challenges?
  - What have you found to be successful in overcoming those challenges?
  - Did the HV offer any advice to help?
- Are you aware of anything that made it easier for you to apply what you had learned from the health visit?
- Was there anything that prevented you from applying what you had learned from the health visit?
  - Probe: A particular diet?
  - Feeding times/habits?
  - Family interference e.g., grandparents?
- Has your child visited a dentist before? If not, is this because you experienced barriers to access?
- Did you have any support from the health visitor in referring you to an NHS dentist? How was your experience?
- Is there any support regarding your child’s oral health that wasn’t offered to you that you would have liked to have?
- Have you incurred any additional costs as a result of wanting to continue with the behaviours from the intervention either for your child or for the family?
  - Probe: Needing to buy more toothpaste or brushes now or perhaps in future? Or even different toothpastes for different family members?
- How do you feel the telephone/ video call consultations went as compared with face-to-face consultations?
  - Probe: Were they as engaging?
- How was the questionnaire you filled in?
  - Probe: Was it too long?
  - How was the wording of the questions?

**Closing:**

Participants will be asked if they would like to add any further information.

**TURN OFF AUDIO RECORDER**

- Thank participants for the discussion and de-brief them on the next steps of the research process.
- Remind the participant their name and contact details used to contact them for this interview will be destroyed after the interview
- Collection of email address or postal address for sending £10 voucher- remind the participant you are storing it in a secure location, and it will destroyed it after we have sent the voucher.

1. Health visitors

**First Dental Steps Intervention - Feasibility Study**

Interview Topic Guide - Health Visitors

*Flexibility should be used when undertaking the interviews and applying the topic guide in terms of wording of questions, order of questions, use of probes/prompts and every opportunity made to allow participants to raise their own issues.*

**Opening:**

- Thank you for participating.
- Few things to run through before we start:
  - Confidentiality – information collected during the study is confidential and access will be restricted to our research team. Some of your comments may be included in a report on the study or in articles for scientific journals but these will be completely anonymous.
  - There are no right or wrong answers, it is your opinion that is valued.

**TURN ON AUDIO RECORDER**

- Consent.
- How do you feel about oral health advice being included in routine health visits?
  - Probe: Do you think it is a priority for your team and parents at the moment?
- Have you received oral health training before the training you received as part of the FDS Intervention?
- What did you think of the content of the training session?
  - Probe: Do you think it covered all of the important issues regarding oral health?
  - Do you think it updated your previous knowledge?
  - Regarding the oral health education you received, do you feel you would have preferred it to be tailored and more specific to the communities you work with?
- What did you think of the actual delivery of the training session?
  - Probe: Did you feel you were able to ask questions and if so, were they answered to your satisfaction?
- What did you find most useful about the training?
- How do you feel about being involved in the dental referral process (to the local Community Dental Service)?
  - Probe: Did you feel you were provided with the appropriate information and support that would enable you to refer families to primary NHS dental services?
  - Were you aware of specific NHS dental services which reduce barriers to dental care including interpreting services and dental services for children with additional needs?
- What was your experience of implementing the intervention into health visits?
  - Probe: How did you fit it into your usual format of the visits?
  - Did it change the way you talk to parents about oral health?
  - Did you link oral health advice to any of the other advice you gave? E.g., healthy eating? Other areas such as managing the child’s behaviour?
  - Was there anything you said which parents received positively or negatively?
  - How did the intervention appear to work for different groups of parents? E.g., those living in the most deprived areas, those who are vulnerable.
- Were there any issues that you feel prevented you from implementing the intervention or made it challenging?
  - Probe: Do you feel you had sufficient time during the visit to discuss oral health?
- Were there any issues that you feel made it easier for you to implement the intervention?
- What are your thoughts on the timing of the intervention?
  - Probe: Do you think it was implemented at an appropriate time for the families and children?
  - How long did it take you to cover OH information in line with FDS intervention?
- Roughly how much time (in minutes) did you used to spend talking about oral health before you started FDS?
- With the FDS intervention, how much time does it take you now to provide the oral health advice?
- Does this make the visit itself last longer? If so, by how long?
- In practice, what questions do parents often ask you about oral health?
- Could you tell me a little bit about the key oral health messages that you advised parents on?
- How confident do you feel about delivering oral health information to parents and answering their questions.
  - Probe: Do you think that the training affected your confidence in your ability to engage in discussions with parents about oral health?
- In your experience, are parents aware of when children should brush their teeth?
- What do you think of the oral health packs?
  - Probe: the content? The aesthetics? How were they received by parents?
- How do you feel the telephone/ video call consultations went as compared with face-to-face consultations?
  - Probe: Were they as engaging?
- How do you think the intervention could be improved?
  - Probe: Training, delivery, timing
- Was there any further support that you feel could have helped you that you did not receive?
- How did you find taking part in the study?
- How did you feel when talking to parents about research and obtaining consent?
- How did you feel about the completion of the questionnaires (digital/paper)?
- How much time did it take to cover study specific details such as the PIS, consent and questionnaire?

**Closing:**

Participants will be asked if they would like to add any further information.

**TURN OFF AUDIO RECORDER**

Thank participants for the discussion and de-brief them on the next steps of the research process.

1. Stakeholders (health visiting team leads, Local Authority oral health leads, and training providers)

**First Dental Steps Intervention - Feasibility Study**

Interview Topic Guide - Stakeholders

*Flexibility should be used when undertaking the interviews and applying the topic guide in terms of wording of questions, order of questions, use of probes/prompts and every opportunity made to allow participants to raise their own issues.*

**Opening:**

- Thank you for participating.
- Few things to run through before we start:
  - Confidentiality – information collected during the study is confidential and access will be restricted to our research team. Some of your comments may be included in a report on the study or in articles for scientific journals but these will be completely anonymous.
  - There are no right or wrong answers, it is your opinion that is valued.

**TURN ON AUDIO RECORDER**

- Consent.
- How do you feel about oral health being included in the Healthy Child Programme generally?
  - Probe: How do you feel about oral health advice being included in routine health visits?
  - Do you think it’s a priority for HVs and parents at the moment?
- How does the intervention fit with the requirements of the national service specification for the Healthy Child Programme (HCP) and specifically with the oral health section?
- In ‘comparison’ areas how is the mandated oral health part of the HCP fulfilled? How would this differ from FDS?

Thinking about the components of the FDS intervention:

Training:

- Do you know of any oral health training available for health visitors other than the training provided as part of the FDS study?
- How did you find the training provided in the FDS?
  - Probe: Cost? Duration? Quality?
- How did the organisation and delivery of the training compare to other training health visitors receive? How was attendance at the training sessions?
- How do you think the training could be improved?

Home visits – health visitors providing advice:

- What are your thoughts on the timing of the oral health advice?
  - Probe: Do you think it delivered at an appropriate time for the families and children?
- How do you think it fits with the other topics covered during the visits?
- Are there any issues that you feel would have made it easier for Health Visitors to provide advice?
- Are there any issues that you feel made it challenging for health visitors to provide advice?
  - Probe: Time? Cost?
- Do you think it made the visits longer for health visitors/parents?
- How was inclusion of oral health advice during the visits monitored?

Home visits – oral health packs:

- What do you think of the oral health packs?
  - Probe: The purchasing and costs? Storage? Distribution? Content? The aesthetics?
- How could the packs be improved?
  - Probe: Training, costs, delivery, timing, cups, leaflets
- Was there any further support that you feel could have been provided concerning the packs?
- How was the provision of packs to parents during the visits monitored?

Future use:

- What are your thoughts on continuing this intervention in your area?
  - Probe: Universal or targeted?
- What are your thoughts on rolling out this intervention to other areas? And on a larger scale?
- What do you think could improve the acceptability of the intervention?
- Are there any challenges you feel need to be taken into consideration?
  - Probe: Healthy Child Programme contracts or commissioning arrangements?

Study procedures:

- How did you find being involved in the study?
  - Probe: Feedback from health visitors about their involvement
- How did you feel recruitment of participants went?
- How did you feel the completion of the questionnaires at baseline and follow-up went?
- Did it feel like the time spent was worth it?
- What would you think about a study that randomised health visiting teams to be involved in FDS or not? Would your service participate?

**Closing:**

Participants will be asked if they would like to add any further information.

**TURN OFF AUDIO RECORDER**

Thank participants for the discussion and de-brief them on the next steps of the research process.
